# Bewohner*innen mit Parkinson-Syndrom in der stationären Altenhilfe: Eine Querschnittsbefragung von Wohnbereichsleitungen in Deutschland

**DOI:** 10.1007/s00391-021-01874-y

**Published:** 2021-03-23

**Authors:** Tobias Mai, Ann-Kathrin Ketter

**Affiliations:** 1 Stabsstelle Pflegeentwicklung, Pflegedirektion des Universitätsklinikums, Goethe-Universität Frankfurt, Theodor-Stern-Kai 7, 60590 Frankfurt am Main, Deutschland; 2 Fakultät Soziale Arbeit, Gesundheit und Pflege, Hochschule Esslingen, Esslingen, Deutschland

**Keywords:** Parkinsonerkrankung, Pflegeeinrichtungen, Prävalenz, Langzeitpflege, Pflege, Parkinson’s disease, Residential facilities, Prevalence, Long-term care, Nursing

## Abstract

**Hintergrund:**

Parkinson-Syndrome führen im Krankheitsverlauf zur Pflegebedürftigkeit bei den Betroffenen. Zur Prävalenz der Bewohner*innen mit einem Parkinson-Syndrom in Pflegeeinrichtungen, zu ihrer Versorgungssituation und zur vorhandenen Expertise der Pflegefachpersonen in den Einrichtungen ist wenig bekannt.

**Ziel der Arbeit:**

Die vorliegende Studie untersucht die Prävalenzrate der Bewohner*innen mit einem Parkinson-Syndrom in stationären Pflegeeinrichtungen in Deutschland. Die Arbeit exploriert die Zusammenarbeit verschiedener Akteure, deren Koordination sowie Information und Wissen des Pflegepersonals. Ziel ist es, einen möglichen Bedarf an spezialisierter Pflege in Pflegeeinrichtungen aufzuzeigen.

**Methode:**

Die schriftliche Querschnittsbefragung der Wohnbereichsleitungen von 500 zufällig ausgewählten Pflegeeinrichtungen in Deutschland erfolgte von Januar bis Juni 2020. Der eingesetzte Fragebogen wurde vorab literaturbasiert entwickelt. Die Daten wurden deskriptiv analysiert.

**Ergebnisse:**

Aus 57 Einrichtungen wurden Fragebogen von 85 Wohnbereichen analysiert (Rücklaufquote 11,4 %). Die Prävalenzrate von Bewohner*innen mit einem Parkinson-Syndrom in der stationären Altenhilfe beträgt 13,9 %. Mehr als die Hälfte haben zusätzlich eine Demenzdiagnose (52,8 %). In 26 % der Fälle erfolgen Krankenhausaufenthalte infolge von Sturzereignissen. Eine eindeutige Koordination der Versorgung durch ärztliche oder pflegerische Spezialisten gibt es nicht.

**Diskussion:**

Bewohner*innen mit einem Parkinson-Syndrom in der stationären Altenhilfe sind häufig, und sie zeigen komplexe motorische und nichtmotorische Symptome – auch durch die Komorbidität Demenz. Die Häufigkeit von Sturzereignissen mit Krankenhausaufenthalten und die geringe Anzahl Parkinson-Syndrom-spezifischer Hilfsmittel zeigen, dass das Wissen der Pflege vor Ort gesteigert werden kann. Eine zentrale Koordination und Unterstützung hierzu sollten in der stationären Langzeitpflege etabliert werden.

**Zusatzmaterial online:**

Zusätzliche Informationen sind in der Online-Version dieses Artikels (10.1007/s00391-021-01874-y) enthalten.

## Abstract (en)

**Background:**

The illness trajectory of Parkinson’s disease leads to a higher need for care. Little is known about the prevalence of residents with Parkinson’s disease in nursing homes, the care situation and the expertise of nurses in these institutions.

**Objective:**

This study examined the prevalence rate of residents with Parkinson’s disease in nursing homes in Germany. In addition, from the subjective point of view of nurse managers, the work of carers, the coordination as well as information and knowledge of the nursing staff were explored.

**Method:**

The cross-sectional survey of nurse managers from 500 randomized selected nursing homes in Germany was conducted from January to June 2020. The questionnaire used was developed based on the literature. The data were analyzed descriptively.

**Results:**

Questionnaires from 85 residential wards in 57 care facilities were analyzed (response rate 11.4%). The prevalence rate of residents with Parkinson’s disease was 13.9%. More than a half had a diagnosis of dementia (52.8%). Affected persons received regular treatment and medical consultations. There were no central coordination of care and no specially trained nursing staff.

**Discussion:**

Residents with Parkinson’s disease are a common occurrence and they show complex symptoms and comorbidities. The frequency of falls with hospital stays and the low number of Parkinson-specific assistive technologies showed that the knowledge of nursing staff can be increased. Central coordination and support for this should be established in long-term care.

## Hintergrund

Parkinson-Syndrome zählen zu den häufigsten neurodegenerativen Erkrankungen im Alter. Zwei Drittel aller Betroffenen leiden an einem primären Parkinson-Syndrom. Derzeit wird die Anzahl der Menschen mit M. Parkinson in Deutschland auf etwa 420.000 geschätzt [9]. Der spätere Verlauf der Erkrankung ist durch eine zunehmende Hilfe- und Pflegebedürftigkeit geprägt.

### Parkinson-Syndrome und Pflegebedürftigkeit

Die Erkrankung ist durch die Kardinalsymptome Akinese, Tremor, Rigor und posturale Instabilität gekennzeichnet. Im Verlauf der Erkrankung kommen nichtmotorische sowie kognitive oder psychiatrische Symptome hinzu. Diese Kombination von Symptomen ist das charakteristische Merkmal für eine langzeitstationäre Versorgung in Pflegeeinrichtungen im späten Verlauf der Erkrankung [1, 11, 24]. Das Parkinson-Syndrom hat nach Demenzen das höchste Risiko für erkrankte Personen über 65 Jahre, pflegebedürftig zu werden [22]. Analysen von Versicherungsdaten zeigen für die USA, dass rund ein Viertel, für Deutschland etwa 20 %, aller Betroffenen mit einem Parkinson-Syndrom in Einrichtungen der Langzeitpflege betreut werden [9, 18].

### Situation in stationären Pflegeeinrichtungen

Je nach Region und Einschlusskriterien liegt die Prävalenz von Bewohner*innen mit einem Parkinson-Syndrom in Langzeitpflegeeinrichtungen zwischen 1,8 und 7,7 % [3, 10, 12, 15, 21]. Sie haben motorische Fluktuationen mit teils langen Phasen ausgeprägter Unbeweglichkeit, sog. Off-Phasen [7, 12, 26]. Drei Viertel der Bewohner*innen mit einem Parkinson-Syndrom gelten als kognitiv beeinträchtigt [2, 8, 12]. Die internationale Studienlage zeigt auf, dass Bewohner*innen mit einem Parkinson-Syndrom in Langzeitpflegeeinrichtungen teils unterberücksichtigt sind [2, 6], obgleich etwa rehabilitative Angebote für den Erhalt kognitiver Funktionen bedeutsam wären. Diagnosen passen nicht zum klinischen Erscheinungsbild, und zu wenige Experten sind in die Versorgung eingebunden. Spezialisierte Pflegefachpersonen haben einen deutlichen Einfluss auf die Versorgungsqualität von Menschen mit einem Parkinson-Syndrom in Langzeitpflegeeinrichtungen, etwa hinsichtlich Lebensqualität, Stimmung, Reduktion unerwünschter Ereignisse wie Stürze und Erhalt der Mobilität [14, 25]. Das Wissen des Personals über die Parkinson-Erkrankung sowie die Organisation der Zusammenarbeit mit Neurolog*innen und mit Krankenhäusern haben entscheidende Einflüsse auf die Versorgung in Pflegeheimen [23].

Laut Statistischem Bundesamt gab es 2019 in Deutschland 15.380 stationäre Pflegeeinrichtungen [4]. Spezialisierte Pflegefachpersonen innerhalb der Pflegeteams gibt es nicht, da die in Deutschland angebotene Fortbildung für Pflegefachpersonen zur „Parkinson nurse“ eine Tätigkeit im Akutsetting voraussetzt [5]. International übernehmen Parkinson Nurses zentrale Funktionen in der Bildung des Personals und der Betreuung der Bewohner*innen [23, 25]. Welche Expertise in den Pflegeteams in Deutschland vorliegt, wie ausreichend diese eingeschätzt wird, und ob es eine zentrale Instanz im Kontext Bildung und Koordination der Versorgung gibt, ist unklar.

## Fragestellungen

Mit der vorliegenden Studie soll die Versorgungssituation der Bewohner*innen mit einem Parkinson-Syndrom in der stationären Altenhilfe untersucht und, damit einhergehend, der Bedarf an pflegerischer Expertise und multiprofessioneller Zusammenarbeit exploriert werden. Es wird untersucht, wie hoch der Anteil der Bewohner*innen mit einem Parkinson-Syndrom in stationären Pflegeeinrichtungen in Deutschland ist. Neben der Prävalenzrate sollen weitere Parameter wie Pflegegrad, Alter, Hoehn-Yahr-Stadium und Demenz als Komorbidität ermittelt werden. Ergänzend sollen durch die Einschätzungen der Wohnbereichsleitungen weitere Fragen zu Zusammenarbeit, Koordination, Wissen und Expertise beantwortet werden.

## Methode

In der vorliegenden explorativen Querschnittserhebung wurde ein literaturbasierter und in Pretests überprüfter Fragebogen verwendet [13]. Der Fragebogen enthält neben Häufigkeitsangaben nominal- und ordinalskalierte Fragen zu subjektiven Einschätzungen.

### Datenerhebung

Auf Grundlage einer Adressdatei von mehr als 10.000 stationären Einrichtungen (pflegemarkt.com) wurden 5 % der Institutionen bundesweit in die Befragung eingeschlossen (*n* = 500). Die Auswahl dieser erfolgte stratifiziert nach Gesamtzahl der Einrichtungen pro Bundesland. Innerhalb der jeweiligen Cluster „Bundesland“ wurden die Einrichtungen via Zufallszahl einer Tabellenkalkulationssoftware randomisiert ausgewählt. Die schriftliche Befragung erfolgte im Zeitraum von Januar bis Juni 2020. Die Einrichtungsleitungen erhielten jeweils einen mit einem Code versehenen Fragebogen, mit der Bitte, diesen an alle Wohnbereichsleitungen in ihrer Einrichtung weiterzuleiten, sowie einen frankierten Rückumschlag. Zusammengehörige Fragebogen einer Einrichtung konnten durch den vergebenen Code zusammengeführt werden.

### Datenanalyse

Die Daten wurden mit der IBM Statistiksoftware SPSS 26 verwaltet und deskriptiv analysiert. Die Prävalenz wurde im Verhältnis zur angegebenen Anzahl der Bewohnerplätze berechnet. Als Grundgesamtheit wurden alle Bewohnerplätze der beteiligten Wohnbereiche herangezogen. Für die subjektiven Einschätzungen im Rahmen der nominal- und ordinalskalierten Fragen wurden relative und absolute Häufigkeiten berechnet. Unterschiede zu erwarteten Häufigkeiten wurden mittels χ^2^-Anspassungstest überprüft.

## Ergebnisse

Von 500 angeschriebenen Pflegeeinrichtungen konnten 85 gültige Fragebogen aus 57 Einrichtungen aus 13 Bundesländern in die Analyse eingeschlossen werden (Rücklauf: 11,4 %).

### Prävalenz und Bewohnermerkmale

In 71 von 85 Wohnbereichen werden Bewohner*innen mit einem Parkinson-Syndrom versorgt. Die Anzahl der Betroffenen schwankt hierbei zwischen 1 und 9/Wohnbereich. Aus den Rückmeldungen ergibt sich eine Prävalenzrate von 13,9 % (Tab. 1).

**Table 1:** 

*Anzahl der Einrichtungen*	*n* = 57	–
*Anzahl der Wohnbereiche*	*n* = 85	
Davon mit PD	*n* = 71	
Davon ohne PD	*n* = 14	
*Anzahl der BmPD*	*n* = 197	Prävalenzrate 13,9 %
*Anzahl der Bewohner, insgesamt*	*n* = 1418	

*PD* „Parkinson’s disease“, *BmPD* Bewohner mit Parkinson’s disease

Von den 197 Bewohner*innen mit einem Parkinson-Syndrom sind 79,2 % mit einem primären Parkinson-Syndrom diagnostiziert (Tab. 2). In 8 % der Fälle konnte keine Form des Parkinson-Syndroms angegeben werden. Mehr als die Hälfte der Bewohner*innen mit einem Parkinson-Syndrom sind weiblich (53,8 %). In fast 20 % der Fälle konnte eine Angabe zum Krankheitsstadium nach Hoehn und Yahr gemacht werden. Mehr als 55 % der Bewohner*innen mit einem Parkinson-Syndrom zeigen mit einem Pflegegrad 4 oder 5 schwerste Beeinträchtigungen der Selbstständigkeit. Mit 52,8 % sind mehr als die Hälfte von einer demenziellen Erkrankung betroffen.

**Table 2:** 

	Gültige Wohnbereiche	Anzahl der Bewohner	%
*Bewohner mit einem Parkinson-Syndrom*	71	197	100,0
Davon mit primärem Parkinson-Syndrom	71	156	79,2
Davon mit atypischem Parkinson-Syndrom	71	8	4,1
Davon mit sekundärem Parkinson-Syndrom	71	17	8,6
Davon mit unbekanntem Parkinson-Syndrom	71	16	8,1
*Hoehn-Yahr-Stadium*			
Hoehn-Yahr-Stadium I	71	2	1,0
Hoehn-Yahr-Stadium II	71	3	1,5
Hoehn-Yahr-Stadium III	71	9	4,6
Hoehn-Yahr-Stadium IV	71	8	4,1
Hoehn-Yahr-Stadium V	71	12	6,1
Hoehn-Yahr-Stadium unbekannt	71	163	82,7
*Bewohner mit einem Parkinson-Syndrom und Demenz*	70	104	52,8
Davon mit Parkinson-Demenz	70	45	43,3
Davon mit Alzheimer-Demenz	69	17	16,3
Davon mit vaskulärer Demenz	70	13	12,5
Davon mit Lewy-Body-Demenz	70	3	2,9
Davon mit anderer/nicht näher bezeichneter Demenz	70	26	25,0
*Männliche Bewohner mit einem Parkinson-Syndrom*	71	91	46,2
*Weibliche Bewohner mit einem Parkinson-Syndrom*	71	106	53,8
*Pflegebedürftigkeit*			
Ohne Pflegegrad	70	1	0,5
Pflegegrad 1	70	0	0,0
Pflegegrad 2	70	24	12,5
Pflegegrad 3	70	63	32,8
Pflegegrad 4	70	67	34,9
Pflegegrad 5	70	37	19,3
*Alter der Bewohner mit einem Parkinson-Syndrom*			
Bewohner < 60 Jahre	70	6	3,1
Bewohner 61–70 Jahre	70	16	8,3
Bewohner 71–80 Jahre	70	57	29,7
Bewohner 81–90 Jahre	70	95	49,5
Bewohner > 90 Jahre	70	18	9,4

### Krankenhausaufenthalte, Therapien, Medikamente und Hilfsmittel

Die häufigsten Gründe für Krankenhausaufenthalte bei Bewohner*innen mit einem Parkinson-Syndrom sind Behandlungen infolge von Sturzereignissen (26,1 %), aufgrund eines schlechten Allgemeinzustandes (20,3 %) oder einer Verschlechterung der Parkinson-Symptome (14,5 %; Zusatzmaterial online: Tab. 1). In nahezu 80 % der Wohnbereiche erhalten die Bewohner*innen mindestens einmal pro Woche eine Physiotherapie (Tab. 3). In 11 Wohnbereichen kann keine Krankengymnastik wahrgenommen werden. Ergotherapie oder Logopädie wird in weniger als der Hälfte angeboten. In einzelnen Fällen besteht die Möglichkeit, eine Psychotherapie wahrzunehmen. Einige Wohnbereiche ermöglichen es den Bewohner*innen, Angebote der Physio- oder Ergotherapie (24 % resp. 13 %) auch ohne ärztliche Verordnung zu nutzen.

**Table 3:** 

	Gültige	Einmal/Woche		Mehrmals/Woche		All 2 bis 3 Wochen		Einmal/Monat		Gar nicht	
		*n*	%	*n*	%	*n*	%	*n*	%	*n*	%
Physiotherapie	68	20	29,4	32	47,1	4	5,9	1	1,5	11	16,2
Ergotherapie	68	14	20,6	12	17,6	2	2,9	1	1,5	39	57,4
Logopädie	68	19	27,9	3	4,4	0	0,0	0	0,0	46	67,6
Psychotherapie	67	1	1,5	1	1,5	0	0,0	2	3,0	63	94,0

Mehr als die Hälfte der Bewohner*innen nehmen mehr als 3‑mal täglich Medikamente zu sich (Zusatzmaterial online: Abb. 1). Nahezu ein Fünftel der Betroffenen benötigt mindestens 6‑mal täglich Medikamente. Nur eine Freitextangabe wurde zu einer speziellen Pumpentherapie gemacht.

In der Nutzung von Hilfsmitteln stehen Rollatoren, Rollstühle und Inkontinenzmaterial im Vordergrund (Tab. 4). Spezielle Hilfsmittel wie Hüftprotektoren, spezielles Essbesteck, Greifzangen oder Anti-Freezing-Stöcke werden kaum benannt.

**Table 4:** 

	Gültig	Anzahl	%
Rollstuhl	71	55	77,5
Rollator	71	53	74,6
Inkontinenzmaterial	71	52	73,2
Duschstuhl	71	42	59,2
Trinkhilfen	71	26	36,6
Lifter	71	18	25,4
Aufstehhilfe	71	16	22,5
Spezielles Essbesteck	71	9	12,7
Hüftprotektoren	71	7	9,9
Andere	71	4	5,6
Gehstock	71	3	4,2
Anti-Freezing-Stock	71	0	0,0

### Koordination der Versorgung

Nahezu immer zum therapeutischen Team werden Pflegefachpersonen und Hausärzt*innen gezählt. Neurolog*innen und Wohnbereichsleitungen gehören in 80 % der Fälle dazu. Nach Einschätzung der Wohnbereichsleitungen werden die therapeutischen Teams sehr unterschiedlich bis gar nicht koordiniert (Tab. 5).

**Table 5:** 

	Anzahl	%
Pflegefachperson	17	24,6
Wohnbereichsleitung	14	20,3
WBL und PF	13	18,8
Es gibt keine Koordination	4	5,8
Hausarzt	3	4,3
Neurologe	3	4,3
WBL, HA, N	3	4,3
WBL, PF, HA, N	2	2,9
WBL, PF, HA	2	2,9
WBL, PF, N	2	2,9
WBL und HA	1	1,4
WBL und N	1	1,4
WBL, PN, Therapeuten	1	1,4
WBL, PF, HA, Phy, Psy	1	1,4
HA, N, Psycho	1	1,4
PF und HA	1	1,4

*PF* Pflegefachperson, *WBL* Wohnbereichsleitung, *HA* Hausarzt, *N* Neurologe, *PN* „Parkinson nurse“, *Phy* Physiotherapie, *Psy* Psychologie

Pflegefachpersonen übernehmen zu 25 % die Koordination des therapeutischen Teams. In weniger als 20 % der Fälle übernehmen diese Rolle die Hausärzt*innen oder die Neurolog*innen. In 40 % der Fälle übernehmen verschieden Akteure zusammen diese Aufgabe, wobei dies in der Mehrzahl die Wohnbereichsleitung und die Pflegefachperson sind. Die Zusammenarbeit mit allen Beteiligten wird von 75 % der Befragten als gut oder sehr gut bewertet. In 88 % der Fälle haben die Bewohner*innen regelmäßigen Kontakt zu Neurolog*innen. Davon haben 54 % einmal im Quartal und mehr als ein Drittel sogar monatlich (38 %) eine fachärztliche Konsultation.

### Pflegeexpertise

Keine der Einrichtungen verfügt über Pflegefachpersonen mit einer Weiterqualifizierung zur Parkinson Nurse (Tab. 6). Das Tätigkeitsfeld einer Parkinson Nurse ist nur 20 % der Wohnbereichsleitungen bekannt. Die Präsenz einer spezialisierten Pflegefachperson wird von 65 % für wichtig bis sehr wichtig eingeschätzt.

**Table 6:** 

	Wohnbereiche *ohne* Bewohner*innen mit einem Parkinson-Syndrom					Wohnbereiche *mit* Bewohner*innen mit einem Parkinson-Syndrom					Alle Wohnbereiche				
	Ja		Nein		Gesamt	Ja		Nein		Gesamt	Ja		Nein		Gesamt
	*n*	%	*n*	%	*n*	*n*	%	*n*	%	*n*	*n*	%	*n*	%	*n*
Ist das Tätigkeitsfeld einer Parkinson Nurse bekannt?	3	21,4	11	78,6	14	12	16,9	59	83,1	71	15	17,6	70	82,4	85
Gibt es eine ausgebildete Parkinson Nurse im Wohnbereich?	0	0,0	14	100,0	14	0	0,0	71	100,0	71	0	0,0	85	100,0	85
Finden mind. einmal im Jahr Schulungen zum Parkinson-Syndrom statt?	1	7,1	13	92,9	14	14	20,3	55	79,7	69	15	18,1	68	81,9	83
Kennen Sie die Weiterbildung zur Parkinson Nurse?	2	14,3	12	85,7	14	14	20,0	56	80,0	70	16	19,0	68	81,0	84
Kennen Sie die Weiterbildung zur PASS?	1	7,1	13	92,9	14	7	10,3	61	89,7	68	8	9,8	74	90,2	82

*PASS* Parkinson-Assistentin

Der Schulungsbedarf zur Pflege von Bewohner*innen mit einem Parkinson-Syndrom wird von etwa der Hälfte der Wohnbereichsleitungen als hoch bis sehr hoch eingeschätzt (51,2 %). Einen Unterschied zwischen Einrichtungen mit und ohne vom Parkinson-Syndrom betroffenen Bewohnern gibt es nicht (χ^2^ = 0,156, df = 1, *p* = 0,693). Die Wohnbereichsleitungen, die keinen Bedarf an Schulungen sehen, verweisen in Freitexten auf die Inhalte der Grundausbildung in der Pflege sowie die teils geringe Anzahl an Betroffenen im Wohnbereich.

## Diskussion

Die Prävalenz von Bewohner*innen mit einem Parkinson-Syndrom in Langzeitpflegeeinrichtungen in Deutschland liegt nach der vorliegenden Befragung mit 13,8 % signifikant höher als in internationalen Vergleichserhebungen (1,8–7,7 %; χ^2^ = 76.517, df = 1, *p* < 0,001) [3, 10, 12, 15, 21]. Die Tatsache, dass in der durchgeführten Studie möglicherweise eher Wohnbereiche mit vom Parkinson-Syndrom Betroffenen teilgenommen haben, führt zur vorsichtigen Interpretation der ermittelten Prävalenz. Bei etwa 820.000 Pflegebedürftigen 2017 in stationären Pflegeeinrichtungen würden demnach ca. 113.000 Bewohner*innen mit einem Parkinson-Syndrom in Pflegeheimen in Deutschland leben. Damit wird in Deutschland, wie in anderen Studien herausgearbeitet, nahezu ein Viertel aller vom Parkinson-Syndrom Betroffenen in stationären Pflegeeinrichtungen versorgt [7, 9, 18].

Nach Hosking et al. weisen 64 % der stationär versorgten Parkinson-Syndrom-Betroffenen ein Hoehn-Yahr-Stadium von 5 auf [12]. Da in den meisten Fällen der vorliegenden Befragung keine Angaben zum Hoehn-Yahr-Stadium gemacht wurde, lässt sich nur durch den Vergleich mit der zu 55 % vorliegenden schweren und schwersten Pflegebedürftigkeit nach den Pflegeraden 4 und 5 ein ähnlicher Stand für Deutschland feststellen. Rund 9 % der Bewohner*innen mit einem Parkinson-Syndrom stürzen. Auch wenn hier nur die Stürze mit Verletzungen resp. Folgen für stationäre Behandlungen gezählt werden können, ist diese Prävalenzrate signifikant höher (χ^2^ = 196.174, df = 1, *p* < 0,001) als generell bei Pflegeheimbewohner*innen (5,5 %) [20]. Nach Zimmermann et al. zeigen Heimbewohner*innen mit kognitiven Beeinträchtigungen ein erhöhtes Risiko zu stürzen [27]. Da mehr als die Hälfte der Bewohner*innen mit einem Parkinson-Syndrom Demenzerkrankungen aufzeigen, scheint dies eine mögliche Erklärung für die hohe Prävalenz für Stürze mit Verletzung in der vorliegenden Untersuchung zu sein. International werden kognitive und psychiatrische Symptome ebenfalls in mehr als der Hälfte der Betroffenen in Pflegeeinrichtungen beobachtet [2, 8, 12]. Damit sind die Versorgungs- und Betreuungssituationen bei Bewohner*innen mit einem Parkinson-Syndrom in der stationären Altenhilfe durch das komplexe Set an motorischen, nichtmotorischen, kognitiven und psychiatrischen Symptomen gekennzeichnet.

Im Kontext der komplexen Symptomatik und Komorbidität sind die therapeutischen Regime zu betrachten. Nahezu 60 % der Bewohner*innen mit einem Parkinson-Syndrom nehmen mehr als 3‑ bis 8‑mal täglich Medikamente ein. Nach Angaben der Wohnbereichsleitungen nehmen 9 Bewohner*innen keine Medikamente ein. Ob diese Entscheidung aufgrund von Unverträglichkeiten bewusst getroffenen wurde oder gar Ausdruck der von anderen Studien aufgezeigten Unterberücksichtigung der vom Parkinson-Syndrom Betroffenen in stationären Pflegeeinrichtungen ist [2, 6], lässt sich mit den vorliegenden Daten nicht klären. Da die medikamentöse Therapie bei Parkinson-Syndromen sehr heterogene Behandlungsmöglichkeiten aufweist, sollte diese mit einer differenzierten Fragestellung bei Bewohner*innen in Langzeitpflegeeinrichtungen etwa im Kontext von Leitlinien näher untersucht werden. Fast 80 % der Wohnbereiche verfügen über physiotherapeutische Angebote, mindestens einmal pro Woche. Während generell etwa 35 % der Betroffenen mit einem Parkinson-Syndrom eine Physiotherapie erhalten [9], scheint das Angebot im stationären Bereich bedarfsorientierter zu sein [2, 6]. In 11 Wohnbereichen mit Bewohner*innen mit einem Parkinson-Syndrom besteht jedoch kein physiotherapeutisches Angebot.

Pflegefachpersonen mit speziellen Weiterqualifizierungen sind kaum bekannt und sind nicht vor Ort eingesetzt. Schwergrade der Erkrankungen, gemessen an den Hoehn-Yahr-Stadien sind der Pflege weitgehend unbekannt; spezielle Hilfsmittel werden kaum eingesetzt. Dennoch besteht seitens der befragten Wohnbereichsleitungen Uneinigkeit, inwiefern Schulungen resp. spezialisierte Pflege sinnvoll in der Versorgung der Bewohner*innen mit einem Parkinson-Syndrom sind. Da die Bewohner*innen eher schwerer betroffen sind und komplexe Krankheitsbilder aufzeigen, ist davon auszugehen, dass eine Expertise innerhalb des Pflegeteams zur Optimierung der Versorgung dieser Gruppe beitragen kann [11, 14, 23]. International nimmt die Parkinson Nurse eine entscheidende und konstante Rolle in einem multiprofessionellen Team ein [17]. In der vorliegenden Befragung zeigt sich keine klare Zuordnung, wer innerhalb des therapeutischen Teams die Koordination der zahlreichen Unterstützungsmaßnahmen übernimmt. Die Parkinson Nurse oder auch Parkinson-Assistentin (PASS) adressiert eher den akutstationären oder ambulanten Sektor in Deutschland [16]. Eine Ausweitung des Wirkungskreises dieser Rollen auf den langzeitstationären Bereich sowie eine verbesserte Zusammenarbeit mit dem stationären Sektor sind erforderlich [23]. Die weitgehend fehlende pflegerische Expertise unter den teilnehmenden Wohnbereichen bestätigt die Forderung nach einer Fachweiterbildung von Pflegefachpersonen zur Parkinson Nurse – bestenfalls als Hochschulausbildung – um „eine qualifizierte, sektorenübergreifende flächendeckende Unterstützung von Parkinson-Patienten“ zu ermöglichen [19]. Hinsichtlich der hohen Prävalenz von demenziellen Begleiterkrankungen wäre eine breitere Ausrichtung einer solchen Qualifikation auf neurodegenerative Erkrankungen sinnvoll.

## Limitation

Die Rücklaufquote ist durch die im Frühjahr 2020 aufgetretene Coronapandemie und die damit verbundenen Herausforderungen für stationäre Pflegeeinrichtungen stark beeinflusst worden. Insbesondere Wohnbereiche ohne vom Parkinson-Syndrom Betroffene haben unter diesen Umständen möglicherweise von einer Teilnahme abgesehen. Auch wenn im Vergleich mit anderen Untersuchungen plausibel, könnte die hier errechnete Prävalenzrate tatsächlich etwas niedriger sein. Da die Ergebnisse dennoch eine hohe Übereinstimmung mit externer Evidenz aufweisen, sollten sie in die Weiterentwicklung der Versorgungstruktur im langzeitstationären Bereich unbedingt eingebunden werden.

## Schlussfolgerungen

In der Mehrzahl der Einrichtungen können Bewohner*innen mit einem Parkinson-Syndrom von begleitenden Therapien und fachärztlicher Betreuung profitieren. Eine zentrale Koordination der beteiligten Akteure scheint es jedoch nicht zu geben. Eine entscheidende Rolle in der Vermittlung und Vernetzung zwischen den Sektoren und den Akteuren sowie in Bereichen der Edukation von Pflegepersonal in der stationären Altenhilfe können spezialisierte Pflegefachpersonen wie Parkinson Nurses einnehmen. Möglicherweise können hier Strukturen der integrierten Versorgung und der Aktivitäten des Vereins der Parkinson Nurses und Assistent*innen e. V. (http://www.vpna-ev.de) erste Ansatzpunkte für Versorgungsnetzwerke für Betroffene in späten Krankheitsstadien sein, die dann in stationäre Langzeitpflegeeinrichtungen hineinwirken.

## Fazit für die Praxis

- Bewohner*innen mit einem Parkinson-Syndrom in der stationären Altenhilfe sind häufig.
- Die meisten Bewohner*innen erhalten regelmäßig Therapien. Eine einheitliche zentrale Koordination der Therapie und Behandlung findet allerdings nicht statt.
- Krankenhausaufenthalte infolge von Sturzereignissen sind häufig. Spezifische Hilfsmittel finden kaum Anwendung.
- Die Unterstützung von spezialisierten Pflegefachpersonen wie Parkinson Nurses erscheint sinnvoll. Diese sollte aufgrund der Streuung der Betroffenen einrichtungsübergreifend organisiert sein.
- Der Aufbau von vernetzten Strukturen etwa zu Expert*innen aus dem akutstationären Bereich resp. die Entwicklung regionaler Schwerpunktbereiche in der stationären Langzeitpflege können Ressourcen bündeln.

## Supplementary Information





