# Supplementary figures and images for: Residents with Parkinson’s disease in the institutional care: A cross-sectional survey of nursing homes in Germany
Source: Z Gerontol Geriatr. 2021 Mar 23;55(5):399–405. [Article in German] doi: 10.1007/s00391-021-01874-y (PMC9360124; doi:10.1007/s00391-021-01874-y)

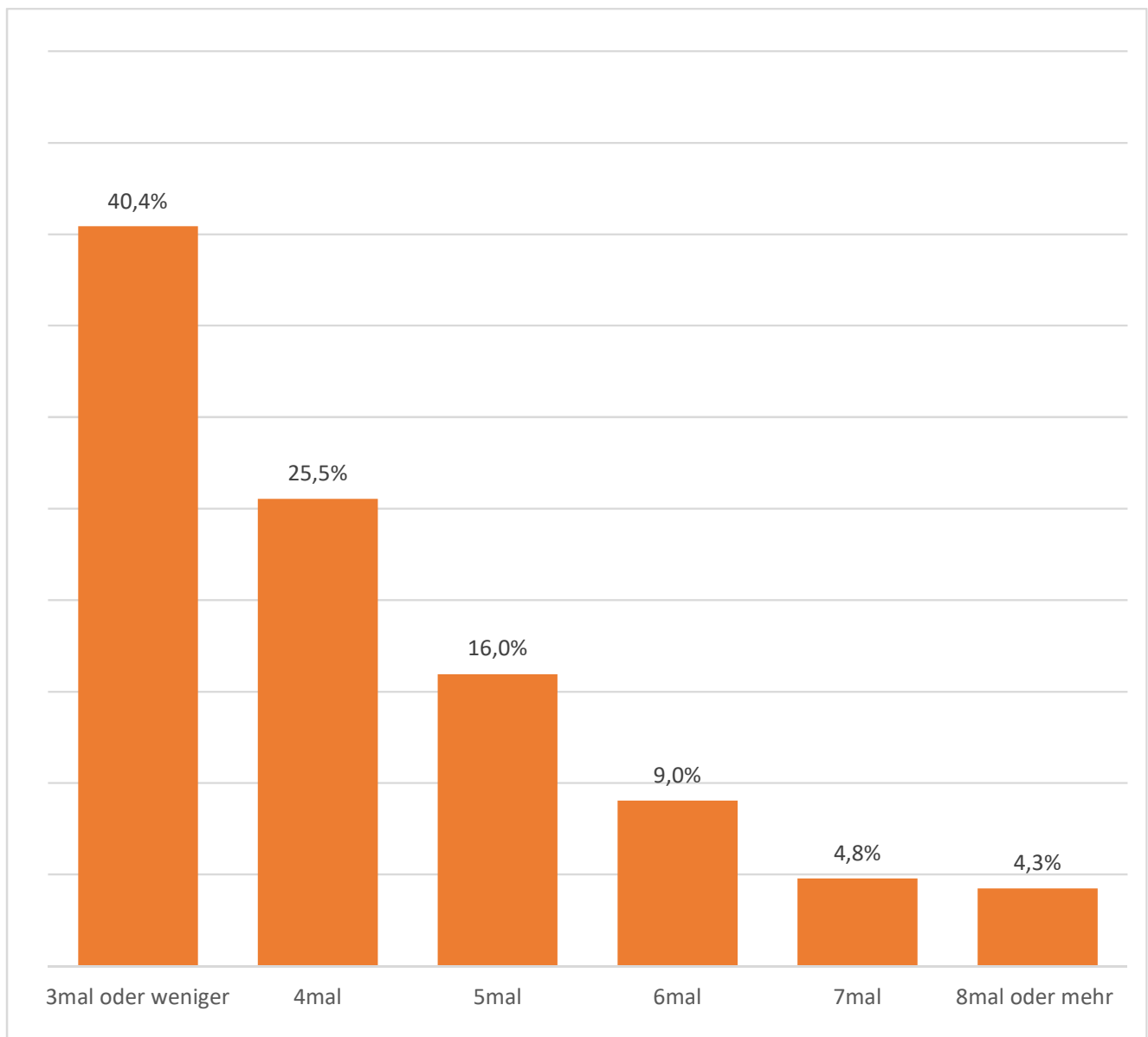

Supplement: Supplementary file 2 [file 391_2021_1874_MOESM2_ESM.pdf]
